# Evaluation of cytotoxicity, immune compatibility and antibacterial activity of biogenic silver nanoparticles

**DOI:** 10.1007/s00430-016-0477-7

**Published:** 2016-09-12

**Authors:** M. Składanowski, P. Golinska, K. Rudnicka, H. Dahm, M. Rai

**Affiliations:** 1 Department of Microbiology, Nicolaus Copernicus University, Lwowska 1, Toruń, Poland; 2 Gastroimmunology Lab., Department of Immunology and Infectious Biology, Institute of Microbiology, Biotechnology and Immunology, Faculty of Biology and Environmental Protection, University of Lódź, Banacha 12/16, Lodz, Poland; 3 Nanobiotechnology Lab., Department of Biotechnology, S.G.B. Amravati University, Amravati, Maharashtra India

**Keywords:** Biogenic silver nanoparticles, Cytotoxicity, NF-κB activation, Antibacterial activity, Actinomycetes

## Abstract

The study was focused on assessment of antibacterial activity, cytotoxicity and immune compatibility of biogenic silver nanoparticles (AgNPs) synthesized from *Streptomyces* sp. NH28 strain. Nanoparticles were biosynthesized and characterized by UV–Vis spectroscopy, transmission electron microscopy, Fourier transform infrared spectroscopy, nanoparticle tracking analysis system and zeta potential. Antibacterial activity was tested against Gram-positive and Gram-negative bacteria; minimal inhibitory concentration was recorded. Cytotoxicity was estimated using L929 mouse fibroblasts via 3-(4,5-dimethylthiazol-2-yl)-2,5-diphenyltetrazolium bromide test. Biocompatibility of AgNPs was performed using THP1-XBlue™ cells. Biogenic AgNPs presented high antibacterial activity against all tested bacteria. Minimum inhibitory concentration of AgNPs against bacterial cells was found to be in range of 1.25–10 μg/mL. Silver nanoparticles did not show any harmful interaction to mouse fibroblast cell line, and no activation of nuclear factor kappa-light-chain-enhancer of activated B (NF-κB) cells was observed at concentration below 10 µg/mL. The half-maximal inhibitory concentration (IC_50_) value was established at 64.5 μg/mL. Biological synthesis of silver can be used as an effective system for formation of metal nanoparticles. Biosynthesized AgNPs can be used as an antibacterial agent, which can be safe for eukaryotic cells.

## Introduction

Nanotechnology deals with the development and utilization of structures and devices with organizational features at the intermediate scale between individual molecules and about 100 nm. Attention is pointed for synthesis of nanoparticles (NPs) from noble metals like gold, silver, zinc or platinum with further applications in science and life [1]. Synthesis of nanoparticles by biological systems is becoming popular. Biosynthesized nanoparticles are less toxic to chemical equivalent and highly stable because of presence of proteins and cost-effective [2]. This leads many researchers to discover new methods for synthesis of nanoparticles by using organisms such as bacteria, fungi or plants. Silver nanoparticles (AgNPs) have gained attention due to antibacterial activity including multidrug resistance (MDR) microorganisms [1, 3] and *Mycobacterium tuberculosis* [4]. Biogenic silver nanoparticles have also shown activity against viruses such as human immunodeficiency virus type 1 (HIV-1) or *Herpex* sp. [5] or antileukemia [6] and anticancer [7] properties.

However, together with broad applications, there is increasing concern related to the biological impacts of use of silver nanoparticles on a large scale and the possible risks to the environment and health. Nanometer size of nanoparticles allows them to easily enter to cells of living organisms, leading to various cell injuries [8, 9]. The potential cytotoxicity and genotoxicity of nanoparticles have risen significantly and have been studied intensively [10, 11]. One of the mechanisms responsible for induction of cytotoxic effect of AgNPs is reactive oxygen species (ROS) formation [12]. ROS may cause harmful effects both to human and to animals as well as in the environment. In this regard, there is increasing interest in the analysis of potential nanoparticle cytotoxicity. Moreover, since nanoparticles may affect not only the cells that compose cellular barriers (fibroblasts, endothelial or intestinal cells) but also the cells of the immune system; it is crucial to define the immunocompatibility of nanoparticles, especially those with potential medical application. The cells especially affected by the foreign particles such as bacteria, viruses and any other external stimuli are the immune cells of the innate immunity such as natural killer cells (NKs), dendritic cells (DC) and monocytes [13]. The latter are very sensitive immune indicators of cell activation, and they sense antigens by Toll-like receptors (TLRs). When recognized, an antigen induced a cascade of signal transmission, which ends with the nuclear factor kappa-light-chain-enhancer of activated B cells (NF-κB) transcription factor activation, resulting in cytokine production such as interferon (IFN)-γ or interleukin (IL)-12 [14]. Those soluble molecules alarm other immune cells and initiate the inflammation process, which leads to a numerous harmful consequences.

The main aim of present work was the synthesis of silver nanoparticles from *Streptomyces kasugaensis* strain NH28 with special reference to antibacterial activity as well as cytotoxic activity and immunosafety on in vitro models.

## Materials and methods

### Isolation of actinobacteria from acidic soil

The actinobacterial strain NH28 was isolated from humic layer of pine forest soil near Torun, Poland (52°55′37″N, 18°42′11″E) on starch casein agar [15] at pH 4.5 using serial dilution method detailed by Golinska [16]. The average pH value of soil was estimated at 3.65 (±1.2). The strain was maintained on starch casein agar slopes at 4 °C.

### Identification of actinobacterium

Strain NH28 was identified based on sequence of 16S rRNA gene. The DNA extraction, PCR-mediated amplification and sequencing were carried out as described previously [16]. The closest phylogenetic neighbors based on 16S rRNA gene similarities were found using the EzTaxon server [17].

### Biosynthesis of silver nanoparticles from NH28 strain

Actinobacterial strain was inoculated in flask with ISP2 broth and incubated in rotatory shaker (150 rpm) at 27 °C for 14 days. After incubation, the biomass was separated by centrifugation at 6000×*g*, washed three times with sterile distilled water, resuspended in sterile distilled water and incubated for 48 h at room temperature. The biomass was then harvested by centrifugation at 6000×*g* for 15 min. The supernatant was collected and filtrated through sterile 0.22-µm filter and treated with 1 mM silver nitrate (AgNO_3_) solution 1:1 (v/v). The reaction mixture was kept in rotatory shaker (130 rpm) at 27 °C in darkness up for 72 h. During the incubation period, flasks were observed for color change of solution from yellow to dark brown.

### UV–visible spectroscopy

Biosynthesis of AgNPs was confirmed by recording of absorption spectra within the range of 200–700 nm using NanoDrop 2000 spectrophotometer (Thermo Scientific, USA).

### Fourier transform infrared (FTIR) spectroscopy

The FTIR characterization of functional groups of silver nanoparticle surface was examined using Spectrum 2000 spectrophotometer (PerkinElmer). The sample was prepared by dispersing the nanoparticles in a matrix of dry potassium bromide (KBr) compressed to form a disk. The spectra were measured in the range of 4000–500 cm^−1^ at a resolution of 4 cm^−1^.

### Transmission electron microscopy (TEM) study

TEM analysis of silver nanoparticles was carried out using the FEI Tecnai F20 X-Twin TEM microscope, at 100 kV. The sample containing dried nanoparticles was diluted with sterile, double distilled water, applied on carbon-coated cooper TEM grids (400 µm mesh size) and then dried in room temperature prior to measurements. The obtained data were evaluated by Statistica Software (StatSoft, USA).

### Nanoparticle tracking analysis (NTA)

The size and size distribution of synthesized nanoparticles were measured using Nanoparticle Tracking Analysis (NanoSight Ltd., Amesbury, UK). Samples were diluted with 5 µL of nuclease-free water, injected into the chamber and measured.

### Zeta potential analysis

The zeta potential was measured using the Zetasizer ZS 90 (Malvern Instrument Ltd, UK). For this purpose, 25 µL of nanoparticle sample was diluted 10 times with water and sonicated for 15 min at 20 Hz to avoid the aggregation of nanoparticles. The sample was then filtered with 0.22-µm filter and used for zeta potential measurement. Measurements were taken in the range of −200 to +200 mV.

### Minimal inhibitory concentration (MIC) of silver nanoparticles from NH28 isolate against bacterial strains

The MIC was determined against bacterial strains such as *Salmonella infantis* (obtained from Sanitary-Epidemiological Station in Torun, Poland), *Proteus mirabilis* (obtained from the collection of microorganisms of the Collegium Medicum in Bydgoszcz, Poland), *Bacillus subtilis* (ATTC 6633), *Staphylococcus aureus* (ATTC 6338), *Klebsiella pneumoniae* (ATTC 700603), *Pseudomonas aeruginosa* (ATTC 10145) and *Escherichia coli* (ATTC 8739). The MICs of synthesized AgNPs were determined by broth dilution method in the 96 microtiter plates (in triplicate). Tryptic Soy Broth (TSB, Becton–Dickinson) was used as diluent for bacterial strains. The final concentration of bacterial cells in each well was 1 × 10^6^ CFU/mL, and concentrations of AgNPs were in the range of 1.25–200 µg/mL. The positive and negative controls were also maintained. After incubation for 24 h at 37 °C, the microtiter plates were read at 450 nm using BIOLOG multimode reader to determine the minimum inhibitory concentration values.

### In vitro biocompatibility

#### Cell cultures

The L929 mouse fibroblasts (LGC Standards, Middlesex, UK) were used for in vitro cytotoxicity testing. The cells were maintained in 25-cm^2^ tissue culture flasks in RPMI 1640 medium (Sigma-Aldrich, St. Louis, MO, USA) supplemented with 10 % fetal bovine serum (FBS) (Cytogen, Lodz, Poland) and antibiotics: 100 U/mL penicillin and 100 µg/mL streptomycin (Sigma-Aldrich, St. Louis, MO, USA) under standard conditions (37 °C, 5 % CO_2_). To obtain a cell suspension for cytotoxicity assay or to start a new culture, confluent monolayers were treated with 0.25 % trypsin solution (Sigma-Aldrich, St. Louis, MO, USA) and their density was evaluated.

THP1-XBlue™ cells derived from the human monocyte THP-1 cell line were used for immunostimulatory assay and maintained in RPMI 1640 medium supplemented with 10 % heat-inactivated FBS (Cytogen, Poland), penicillin: 50 µg/mL, normocin: 100 µg/mL and zeocin: 200 µg/mL (Invitrogen, San Diego, CA) at 37 °C in incubator with 5 % CO_2_ and 95 % air. Cells were re-cultured every 3 days to maintain concentration of <2 × 10^6^ cells/mL and used with <20 passages after thawing.

Both cell culture types were supplemented with fresh medium two or three times per week to maintain their growth in log phase. Prior to experiments, the viability of the cells was assessed by exclusion of trypan blue stain and remained in the range of 93–95 %.

#### Cytotoxicity assay

To evaluate the cytotoxicity of AgNPs, the L929 cells at the density of 2 × 10^5^/mL (100 µL per well) were distributed into the 96-well plates, and then, nanoparticles at final concentration of 1, 5, 10, 25, 50 or 100 µg/mL were added (all in triplicate). Untreated cells constituted a positive control of viability, whereas cells treated with 2 % saponin (Sigma-Aldrich, St. Louis, MO, USA) were used as a positive control of cell lysis. Biogenic AgNPs in culture medium with no cells were included as a internal control of a potential background. Plates were incubated under standard conditions for 24 h. The cell viability was evaluated by the MTT (3-(4,5-dimethylthiazol-2-yl)-2,5-diphenyltetrazolium bromide) colorimetric technique based on the ability of viable cells to reduce MTT (Cell Proliferation and Cytotoxicity Assay, R&D Systems, Minneapolis, MN, USA) to formazan crystals. Briefly, following the 24-h incubation, 10 µL of MTT solution was added to each well. After four hour incubation (37 °C, 5 % CO_2_), the plate was centrifuged (400 g/10 min.), supernatants were removed and the intracellularly stored formazan was solubilized with 200 µL of the dissolving solution for 8 h at room temperature. The optical density was then measured at a reference wavelength of 570 nm using plate reading spectrophotometer Victor 2 (Wallac, Turku, Finland). The half-maximal inhibitory concentration (IC_50_) values were calculated using statistic software GraphPad Prism 5 (USA).

#### Membrane integrity assay

The integrity of cellular membrane of L929 mouse fibroblasts was evaluated using standard trypan blue exclusion assay. The cells were incubated in the 96-well culture plate (1 × 10^5^/well) in the presence of silver nanoparticles in various concentrations (1, 5, 10, 25, 50 and 100 µg/mL) in triplicate for 24 h. Untreated cells were used as negative control (100 % viability), whereas cells treated with 2 % saponin constituted a positive control of membrane disintegration. Following the incubation, the medium was discarded and cells were incubated for 2 min with 4 % trypan blue in culture medium. Assuming that the dye stains only the cells with damaged cell membrane, the percentage of disrupted (dead) and unchanged (viable) cells was estimated using light inverted microscope. The assay was performed in triplicate for each sample and with four technical repeats.

#### Cellular adherence and morphology

The ability of the cells to adherence and to create a tight confluent monolayer was evaluated by the incubation of previously settled L929 fibroblasts with silver nanoparticles in various concentrations. The cells were introduced to a 24-well culture plate (5 × 10^5^/well/mL) and incubated overnight to create a tight monolayer exhibiting complete confluence. Then, AgNPs in various concentrations (1, 5, 10, 25, 50 and 100 µg/mL) were added to cellular monolayers in triplicate and incubated for 24 h in humidified conditions in the presence of 5 % CO_2_. Following the incubation, monolayers were washed twice to remove detached cells and suspended in fresh culture medium. Untreated cells were considered negative control (100 % confluence), whereas cultures treated with 2 % saponin were used as a positive control (induced loss of adherence). To visualize the effect of nanoparticles on the cellular adherence and morphology, the percentage of the cellular confluence was determined by Image J 1.49 image processing software (National Institute of Health, Maryland, USA), and the photographs of representative cultures were taken under the inverted light microscope.

#### Quantification of NF-κB induction

The immunostimulatory effect of the silver nanoparticles in eukaryotic cells was tested using the THP1-XBlue™ cells (Invitrogen, San Diego, CA), which stably express an NF-κB/AP-1-inducible reporter system to facilitate the monitoring of TLR-induced NF-κB/AP-1 activation, resulting in the secretion of embryonic alkaline phosphatase (SEAP). To estimate immunostimulatory effect of AgNPs in various concentrations, the THP1-XBlue™ indicator cells were plated at an initial density of 1 × 10^6^ cells/mL in 96-well tissue culture plates (100 µL/well). To each well, AgNPs in various concentrations (1, 5, 10, 25, 50 and 100 µg/mL) were added and incubated for 24 h under humidified conditions with 5 % CO_2_. The untreated cultures were used as negative controls, whereas cultures treated with lipopolysaccharide (LPS) of *Escherichia coli* (Sigma-Aldrich, St. Louis, MO, USA) at final concentration of 1 µg/mL were used as positive controls. The 20 µL of supernatant from each sample was then transferred to a separate 96-well plate containing 200 µL of Quanti-Blue reagent (InvivoGen, San Diego, CA). The quantification of SEAP as a marker of cell activation was developed after 24-h incubation (37 °C, 5 % CO_2_). The OD values were measured in multifunctional reader Victor 2 (Wallac, Turku, Finland) at the wavelength of 650 nm. Experiments were repeated four times in triplicate for each compound and control.

### Statistical analyses

The differences between values were tested using the Mann–Whitney’s *U* test for impaired data preceded by the evaluation of normality and homogeneity of variances. The results were considered statistically significant when *P* < 0.05. For statistical analysis, the STATISTICA 6.0 PL software was used (Stat Soft, Poland).

## Results

### Identification of actinobacterial strain

Almost complete 16S rRNA gene sequence (1376 nucleotides [nt]) of the strain NH28 was determined. The strain NH28 was related to *Streptomyces kasugaensis* M338-M1^T^ and found to share 100 % 16S rRNA gene similarity.

### Characterization of silver nanoparticles

The color change of cell filtrate from yellow to dark brown after treatment with silver nitrate solution was observed, due to excitation of surface plasmon vibrations of nanoparticles.

**Fig. 1:**
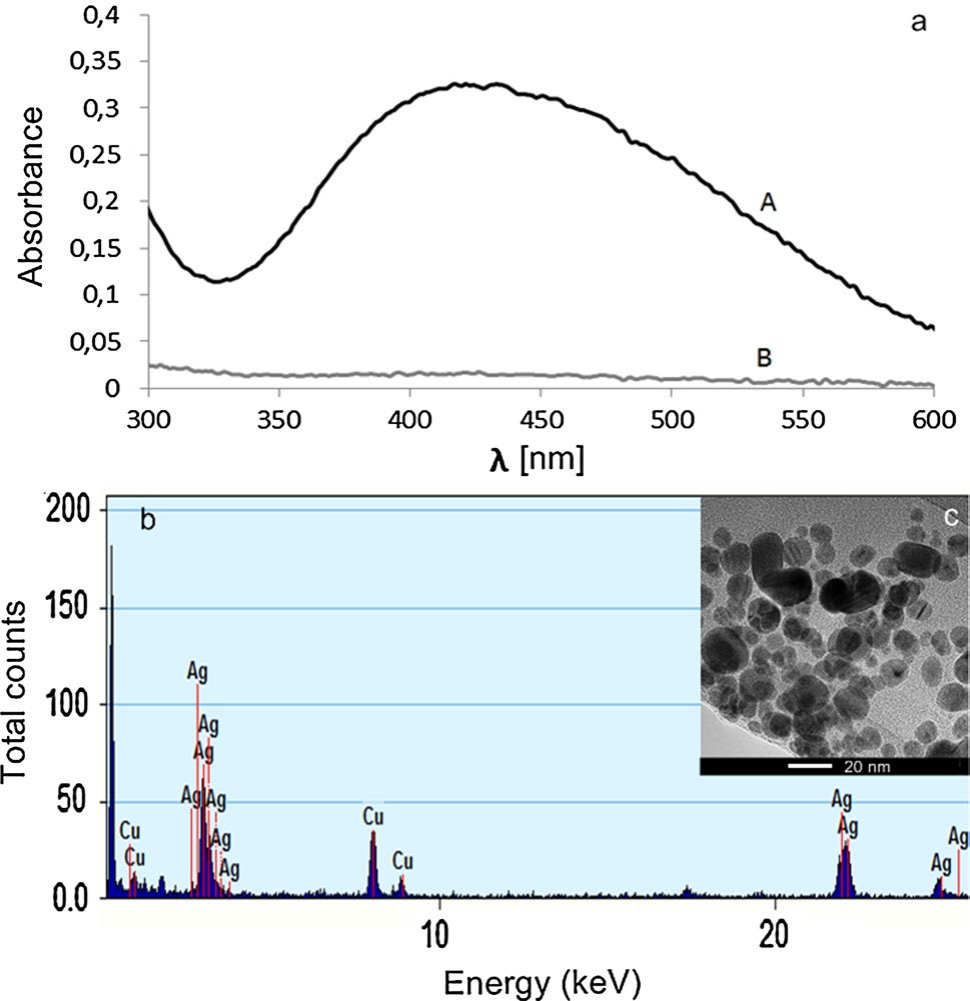
UV–Vis spectrum (**a**), EDX analysis (**b**) and TEM micrograph (**c**) of silver nanoparticles

The presence of biosynthesized silver nanoparticles was confirmed by UV–Vis spectrophotometer analysis, which revealed absorbance peak at wavelength of 421 nm (Fig. 1a), and TEM analysis, which showed polydispersed and spherical in shape nanoparticles with size range of 4.2–65 nm (±9.7 nm), mean size of 19.9 nm (±13 nm). The EDX analysis revealed strong signal of Ag metal in analyzed sample (Fig. 1b, c).

**Fig. 2:**
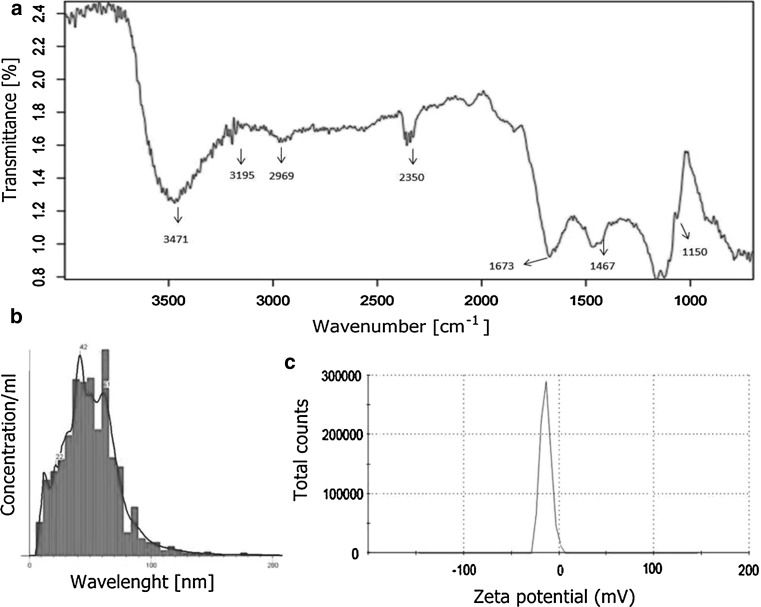
FTIR spectrum (**a**) NTA (**b**) and zeta potential distribution (**c**) of silver nanoparticles

FTIR studies of AgNPs revealed seven peaks at 3471, 3195, 2969, 2357, 1673, 1467 and 1158 cm^−1^, which can refer to possible presence of proteins over the surface of biosynthesized AgNPs (Fig. 2a).

The Nano Tracking Analysis recorded average size of synthesized silver nanoparticles of 52 nm (±25) at the concentration of 0.451 × 10^8^ particles/mL (Fig. 2b).

The stabilization of biosynthesized Ag nanoparticles was examined by zeta potential measurement. The zeta potential value was found to be −13.8 mV (Fig. 2c).

### Antibacterial activity against pathogenic bacteria

The antibacterial activity of silver nanoparticles obtained from *Streptomyces kasugaensis* strain NH28 showed reliable activity against all tested bacterial strains. The highest antibacterial activity of biosynthesized AgNPs was observed against *Staphylococcus aureus*, followed by *Klebsiella pneumoniae*, *Proteus mirabilis* and *Escherichia coli*, then *Bacillus subtilis*, *Salmonella infantis* and at least against *Pseudomonas aeruginosa* (Table 1).

**Table 1:** Minimum inhibitory concentration of AgNPs from NH28 strain against different bacterial pathogens

Tested microorganism	MIC
*Staphylococcus aureus* (ATTC 6338)	1.25 ± 0.078
*Klebsiella pneumoniae* (ATTC 700603)	1.25 ± 0.007
*Proteus mirabilis*	1.25 ± 0.035
*Escherichia coli* (ATTC 8739)	1.25 ± 0.010
*Bacillus subtilis* (ATTC 6633)	2.5 ± 0.025
*Salmonella infantis*	10 ± 0.011
*Pseudomonas aeruginosa* (ATTC 10145)	10 ± 0.025

Values expressed in mean ± SD

The lowest MIC value (1.25 µg/mL) of biosynthesized AgNPs was found against *Staphylococcus aureus*, *Klebsiella pneumoniae*, *Proteus mirabilis* and *Escherichia coli* (Table 1), which inhibited growth of 48, 29, 23 and 14 % bacterial cells, respectively. The MIC of AgNPs against *Bacillus subtilis* was found in concentration of 2.5 µg/mL, whereas against *Pseudomonas aeruginosa* and *Salmonella infantis* of 10 µg/mL (Table 1), which inhibited growth of 8 % bacterial cells.

### In vitro biocompatibility of biogenic silver nanoparticles

The safety of biogenic silver nanoparticles on in vitro model was evaluated by the incubation of AgNPs in various concentrations with L929 fibroblasts and further analysis of their viability (MTT reduction assay), cellular monolayer confluence (light microscope) and membrane integrity (trypan blue exclusion assay).

**Fig. 3:**
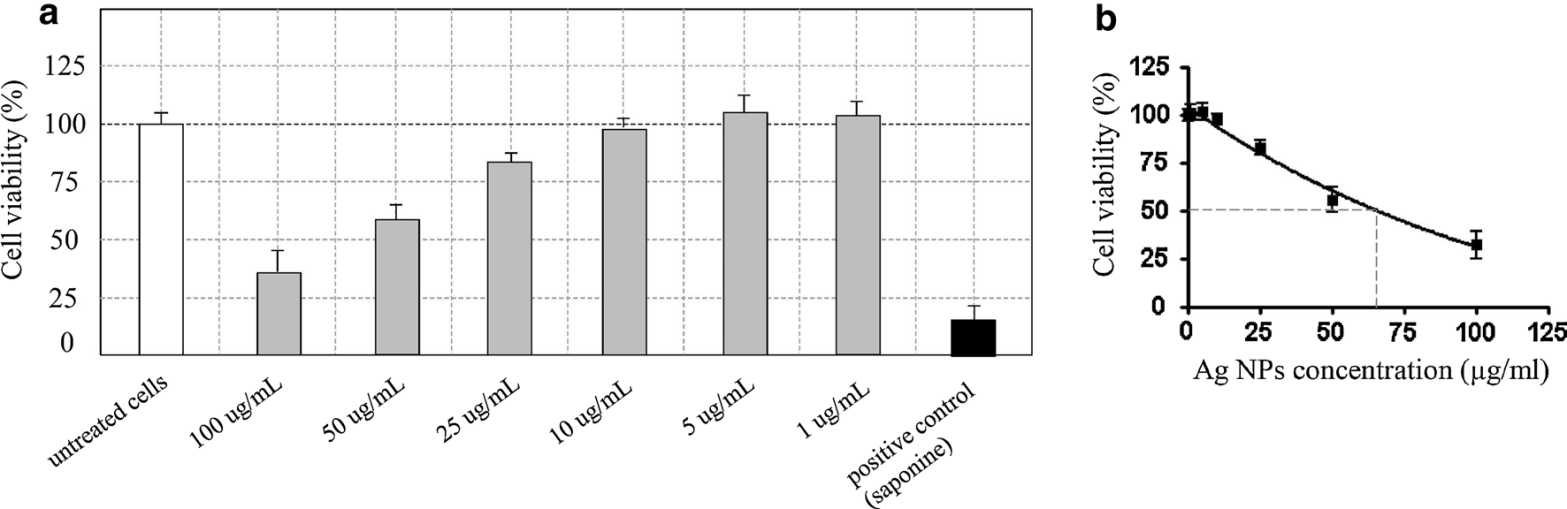
Viability of L929 mouse fibroblasts after treatment (24 h, 37 °C, 5 % CO_2_) with various concentrations of biogenic silver nanoparticles (AgNPs) in comparison with untreated cells (negative control) and cells treated with lytic agent (2 % saponin) evaluated in MTT reduction assay (**a**). The IC_50_ value calculated in GraphPad Prism software on the basis of MTT reduction assay (**b**)

The viability of untreated L929 fibroblasts remained unchanged and constituted 100 %, whereas the cultures treated with 2 % saponin exhibited significantly lower percentage of viable cells (12.6 ± 3.2; *P* = 0.004). Similarly, biogenic AgNPs in concentrations of 100 and 50 µg/mL induced significant reduction in cell viability, in comparison with untreated cultures (31.2 ± 6.5 %; *P* = 0.01) and (55.7 ± 6.1 %; *P* = 0.03) (Fig. 3a). A relatively lower viability was observed in cultures treated with AgNPs in a concentration of 25 µg/mL (82.9 ± 7.5 %), whereas there was no cytotoxic effect observed for concentrations of 1, 5 and 10 µg/mL. The IC_50_ value calculated in GraphPad Prism software was 64.5 µg/mL (Fig. 3b).

**Fig. 4:**
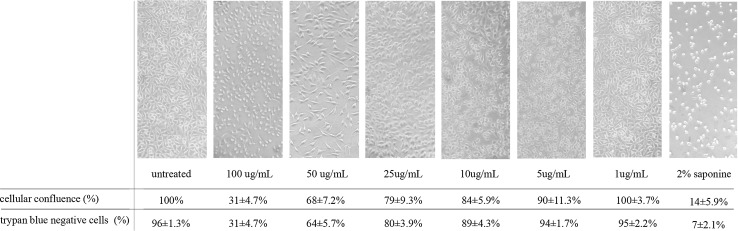
Impact of AgNPs on the confluence of L929 monolayer and membrane integrity measured by trypan blue exclusion assay. The membrane integrity was evaluated by basic trypan blue exclusion assay. The percentages and standard deviations for cells that did not absorb the dye (viable cells) are shown

The results of cytotoxicity were consistent with the observations from trypan blue exclusion assay as well as monolayer confluence experiments. Cellular confluence is expressed as a percentage (%±SD) of cells able to adhere to the surface of a tissue culture plate measured by Image J software and shown on representative photographs from light microscope (40×). The membrane integrity was evaluated by basic trypan blue exclusion assay. The percentages of cells that did not absorb the dye (viable cells with untouched membrane structure) are shown (Fig. 4). The experiments were conducted in triplicate and four technical repeats.

Untreated cultures exhibited a confluent (100 %) tight monolayer, whereas the cultures treated with lysis agent displayed a significant loss of monolayer integrity (14 ± 5.9 %; *P* = 0.004). Similarly, the AgNPs used in concentrations of 50 and 100 µg/mL interfered with cell capacity to adhere, which was observed as a significant decrease in monolayer confluence: 68 ± 7.2 (*P* = 0.04) and 31 ± 4.7 % (*P* = 0.002), respectively. There was no significant negative effect of AgNPs on cellular integrity when used in concentrations of 5 and 10 µg/mL; however, still a reduction in monolayer integrity to 90.0 ± 11.3 % and 84 ± 5.9 %, respectively, was observed. The concentration of 1 µg/mL did not affect the monolayer confluence, which was similar to conditions of cells cultured in complete medium (untreated cells).

The membrane integrity of cells was significantly reduced when concentrations 10, 25, 50 and 100 µg/mL were used (*P* < 0.05), (Fig. 4). In contrast, the cellular membrane remained unchanged, when AgNPs were introduced in concentrations of 1 and 5 µg/mL.

**Fig. 5:**
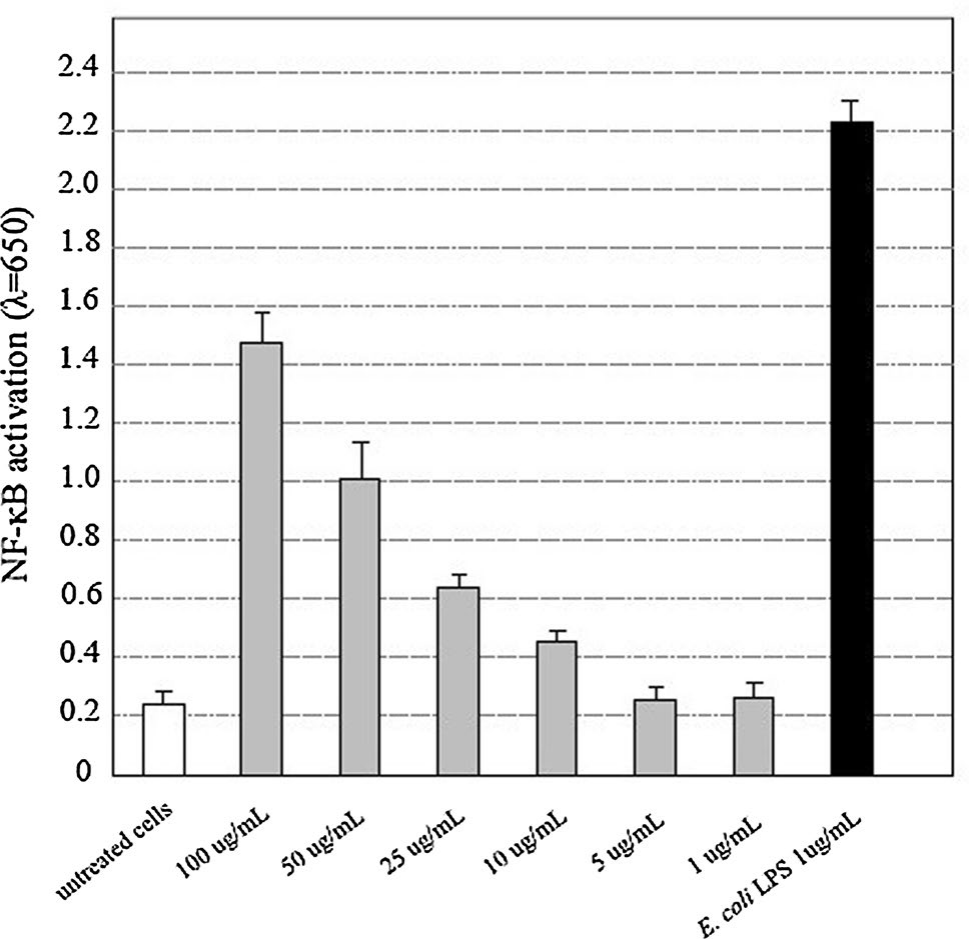
Immunocompatibility of tested biogenic AgNPs evaluated on THP1-XBlue cells. The mean OD values and standard deviations for four technical repeats (in triplicate) are presented in relation to untreated cells (negative control) and cells treated with bacterial lipopolysaccharide (*Escherichia coli*) as a positive control. The OD values were measured at *λ* = 650 nm

Immunocompatibility assay using THP1-XBlue cells showed that concentrations of biosynthesized AgNPs at 10 µg/mL generated activation of human monocyte-macrophage cell line. It was also observed that the response of monocytes to AgNPs was dose dependent (Fig. 5). Well-established bacterial stimuli—*E. coli* lipopolysaccharide—induced strong activation of monocytes, in comparison with untreated cultures (*P* = 0.004). None of the biosynthesized AgNPs induced such high reaction; however, nanoparticles used in concentrations of 100 µg/mL (*P* = 0.01) and 50 µg/mL (*P* = 0.03) significantly upregulated the induction of NF-κB transcription factor responsible for monocyte activation (Fig. 5). There was no such effect in case of THP1-XBlue cells treated with AgNPs in concentration of 1 and 5 µg/mL, and the obtained values remained on the level of untreated cultures.

## Discussion

In our study, we confirmed synthesis of silver nanoparticles from *Streptomyces kasugaensis* strain NH28—visually by the color change of reaction mixture and spectroscopically (UV–Vis spectroscopy) which revealed peak at characteristic wavelength of 421 nm specific for silver nanoparticles. Similar results were reported by Manivasagan [18] or Chauhan [19] who used actinobacteria for synthesis of AgNPs and found related values at approximately 420 nm.

Silver nanoparticles exhibit an intense absorption peak in the visible region 400–440 nm due to the surface plasmon excitation (SPR); this absorption band is observed by combined oscillation of free conduction electrons of metal nanoparticles in resonance with light wave [20].

The size, shape and stability of the biosynthesized AgNPs were characterized by TEM, NTA, FTIR and zeta potential analyses. TEM analysis showed small-sized, polydispersed silver nanoparticles. Comparable results of TEM analysis were recorded by Subashini [21] who observed 20–70 nm and spherical silver nanoparticles biosynthesized by *Streptomyces* sp. VITBT7. However, either smaller or bigger silver nanoparticles have been derived from actinobacteria [22, 23].

Our EDX spectroscopy spectra showed strong signal of Ag with high weight percentage of Ag in tested samples, which confirmed the presence of silver in the sample.

NTA technique tracks particles for number of steps and calculates their size based on the distance travelled. This system offers ability to measure concentration and can be used to give date about size and size distribution [24]. Similar results were recorded by Anasane [25] who observed an average size of AgNPs synthesized from two actinobacterial strains of 30 and 28 nm and its concentration of 6.08 × 10^6^ and 6.16 × 10^8^ particles per mL, respectively.

The FTIR analysis of AgNPs is performed to identify the possible biomolecules responsible for the reduction of Ag ions to AgNPs and to assess stabilized proteins over the surface of nanoparticles as a capping agent [26]. The absorption band at 3471 cm^−1^ can be assigned to O–H stretching vibration, whereas at 3195 and 2969 cm^−1^ to primary and secondary amines, respectively [27, 28]. Peak at 2357 cm^−1^ can be assigned to N–H stretching vibration, at 1673 cm^−1^ to C=C stretching vibration, and 1467 cm^−1^ can be attached to C=C stretching vibrations of an aromatic amine group or C–N stretching vibrations of aromatic amines (Fig. 3) [29]. The presence of band at 1158 cm^−1^ corresponds to C–O stretching vibration [30]. The above observations confirmed the presence of proteins responsible for the reduction process and formation of capping agent on the surface of AgNPs. It has been reported that proteins can bind to nanoparticles either through their free amine groups or through cysteine residues and increase stabilization of the AgNPs. It is also claimed that carbonyl groups of amino acids and peptides have strong ability to bind to silver [31–33].

The zeta potential analysis is used to record stability of synthesized nanostructures. The higher the negative or positive zeta potential, the stronger the prevention of AgNPs from its aggregation [34, 35]. Our results showed negative charge on the surface of biosynthesized AgNPs and its high stability. Similar results were reported by Anasane [25] and Golinska [36] who studied silver nanoparticles from actinobacteria and noticed its zeta potential in the range of −16.6 and −24.1 mV. However, Kaler [34] found less stable mycogenic nanoparticles with charge of −31 mV.

Antibacterial activity of biogenic silver nanoparticles has been proven by many researchers [3, 36–40]. In our study, we recorded high antibacterial activity of silver nanoparticles from *Streptomyces kasugaensis* strain NH28 against Gram-positive (*B. subtilis*, *S. aureus*) and Gram-negative (*S. infantis*, *P. mirabilis*, *K. pneumoniae*, *P. aeruginosa*, *E. coli*) isolates. The MIC values of silver nanoparticles against bacterial strains obtained in this study were low and confirmed high antibacterial activity of NPs. The low values of MIC after treatment bacteria with AgNPs were also found by other authors. Paredes [41] observed the MIC values of AgNPs at 0.25 µg/mL for MRSA and 0.50 µg/mL for *E. coli* O157:H7. Similarly, Zarei [42] determinates low MIC value of biosynthesized AgNPs for *Escherichia coli* O157:H7 and *Salmonella typhimurium*, both at 3.12 µg/mL. In other studies, Ansari [38] estimated the MIC value at 12.5 µg/mL for reference strain *S. aureus* ATCC25923. However, Singh [43] who studied MIC of AgNPs synthesized from *Acinetobacter calcoaceticus* reported activity of AgNPs against human pathogenic bacteria in much higher concentration range of 150–600 μg/mL as compared to our findings.

It is proposed that antibacterial activity of biosynthesized silver nanoparticles is related to interaction of AgNPs with sulfur-containing proteins present in cell membrane. It causes disturbance of its power function, disintegration of membrane structure and release of cell contents [9, 44–46]. It has been reported that antimicrobial activity of silver nanoparticles is related to the formation of free oxygen radicals, which induce cell membrane damage [47]. The cellular proteins, including enzymes and amino acids residues [48] as well as DNA, may be the potential sites for the AgNPs. The possible damage caused by the AgNPs reacting with DNA may affect on cell division and DNA replication and finally leading to the cell death [49, 50]. The silver nanoparticles getting into the bacterial cells can also release silver ions, which may contribute to the antimicrobial activity of metal nanoparticles [18, 19].

It is claimed that the total surface area-to-volume ratios of the nanoparticles affect on its antimicrobial properties. Thus, smaller nanoparticles display higher antibacterial activity when compared to bigger ones [51]. In the present study, we found small-sized (mean 19.3 nm) nanoparticles from *Streptomyces kasugaensis* strain NH28.

Silver nanoparticles can be responsible for release of lipopolysaccharides and membrane proteins, collapsing of bacterial membrane potential induced by drop of ATP level by nano-Ag [52, 53]. Researches propose many mechanisms of AgNPs antibacterial activity, but still exact mechanism has not been confirmed and needs further studies.

In our study, we have aimed to evaluate the cytotoxicity of obtained AgNPs in a direct cytotoxicity assay toward routinely used in such experiments cell line of L929 fibroblasts. We found that silver nanoparticles at concentration 1 and 5 µg/mL did not show any cytotoxic properties and the viability of target cells remained at the same level as untreated controls. The cytotoxic effect was observed, especially for high concentrations of AgNPs, such as 50 µg/mL. The IC_50_ value was established at 64.5 μg/mL. Arora [54] used AgNPs of 7–20 nm and reported that concentrations of 25 and 100 μg/mL did not induce morphological changes compared to control cells, and the IC_50_ value was equal to 61 μg/mL—similar to the value obtained in our study. It has also been reported that smaller-sized AgNPs exhibit high cytotoxic potential than larger-sized nanoparticles [55, 56]. Park (2011) characterized 5-nm particles as more toxic and 20- and 50-nm particles as less toxic even when compared to silver ions. Similarly to our study, research was performed on L929 fibroblast cells line, routinely used in cytotoxicity assays. Barua [57] performed a research on HeLa cells, which revealed that particle size in range of 7–14 nm and concentrations up to 20 µg/mL did not affect the viability of the cells. However, in MTT assay they showed that silver nanoparticles inhibited the HeLa cells proliferation, and this phenomenon was dose dependent. Results presented here shows that biogenic AgNPs obtained by us in a model of actinomycetes exhibit low cytotoxicity confirmed in cytotoxicity assay and by high IC_50_ value. What is more, we have also showed that the doses below 10 µg/mL are not only safe in the context of direct cytotoxicity but also do not affect membrane integrity and the ability of the cells to adhere. Those properties are very crucial in maintenance of cellular barrier function on various levels: epithelial, endothelial or intestinal [58]. Studies confirm that AgNPs as well as silver ions are responsible for inducing toxicity in organisms [59, 60]. Many authors suggested that ROS plays central role in silver nanoparticle-induced cell death [61]. Studies have shown that most common mechanisms of AgNPs toxicity are a result of oxidative stress [62, 63]. Silver nanoparticles generate high intracellular ROS level and decline antioxidant enzymes like GSH, which leads to disconcert functions of cell [54, 62]. AgNPs disrupt normal cellular function, affect the membrane integrity and induce various apoptotic signaling genes of cells, leading to programmed cell death [64]. The cytotoxic effects of silver nanoparticles can also be generated due to active physicochemical interaction of silver atoms with the functional groups of cell proteins [65]. It has been recorded that biogenic nanoparticles caused lesser cytotoxicity compared with silver ions or commercial silver nanoparticles [46].

The immune cells are very sensitive indicators that recognize and rapidly react to foreign antigens such as microbiological, physical and chemical ones. In vitro immunocompatibility assay on THP1-XBlue cells revealed that biogenic silver nanoparticles in concentration of 5 µg/mL or lower do not cause the activation of monocytes. However, concentrations of 50 µg/mL or higher significantly upregulated the monocyte activation. Similarly to our results, Stępkowski [66] reported that NF-κB have been activated after exposure to AgNPs (100 µg/mL), and this effect of action was observed only in HepG2 (human liver carcinoma cell line) cells but not in A549 (non-small cell lung cancer line) cells, which can be induced by antioxidants. The activation mode in many immune cells, including monocytes, is NF-κB-dependent, and if stimulated, the cells secrete inflammatory cytokines, leading to the immune response such as inflammation [67]. Many studies showed that silver nanoparticles can provide an activation signal for NF-κB and induce the production of pro-inflammatory mediators such as TNF-α, IL-8, IL-2 and IL-6 [68, 69]. Shi [70] also noticed that AgNPs could induce the injury and dysfunction of human umbilical vein endothelial cells through the activation of NF-kB, which is associated with oxidative stress, induced by AgNPs. Here we report that biogenic AgNPs obtained in actinomyces model exhibit low cytotoxicity and weak stimulatory potential to activate monocytes—favorable features that will be further investigated. Based on obtained data, we assume that silver nanoparticles can be a promising and safe antibacterial agent.
